# Amino Acid Profiling Identifies Disease-Specific Signatures in IgE-Mediated and Non-IgE-Mediated Food Allergy in Pediatric Patients with Atopic Dermatitis

**DOI:** 10.3390/biomedicines11071919

**Published:** 2023-07-06

**Authors:** Kacper Packi, Joanna Matysiak, Szymon Plewa, Agnieszka Klupczyńska-Gabryszak, Eliza Matuszewska, Natalia Rzetecka, Anna Bręborowicz, Jan Matysiak

**Affiliations:** 1 Department of Inorganic and Analytical Chemistry, Poznan University of Medical Sciences, 60-806 Poznan, Poland; kacperpacki1@wp.pl (K.P.); splewa@ump.edu.pl (S.P.); aklupczynska@ump.edu.pl (A.K.-G.); eliza.matuszewska@ump.edu.pl (E.M.); natalia.rzetecka@student.ump.edu.pl (N.R.); 2 AllerGen, Center of Personalized Medicine, 97-300 Piotrkow Trybunalski, Poland; 3 Faculty of Health Sciences, Calisia University—Kalisz, 62-800 Kalisz, Poland; jkamatysiak@gmail.com; 4 Department of Pulmonology, Pediatric Allergy and Clinical Immunology, Poznan University of Medical Sciences, 60-572 Poznan, Poland; abreborowicz@wp.pl

**Keywords:** food allergy, atopic dermatitis, biomarkers, children, LC-MS/MS, amino acid profiling, component-resolved diagnostics

## Abstract

An IgE-mediated food allergy (FA) in atopic dermatitis (AD) children should be easily differentiated from other immune-mediated adverse effects related to food. Specific IgEs for particular protein components has provided additional diagnostic value. However, component-resolved diagnostics (CRD) has not solved all diagnostic problems either. We analysed the serum profile of 42 amino acids (AAs) in 76 AD children aged 2–60 months with an IgE-mediated FA (*n* = 36), with a non-IgE-mediated FA (*n* = 15) and without an FA (*n* = 25) using high-performance liquid chromatography coupled with mass spectrometry (LC-MS/MS) and an aTRAQ kit. We identified homocitrulline (Hcit), sarcosine (Sar) and L-tyrosine (Tyr) as features that differentiated the studied groups (one-way ANOVA with least significant difference post hoc test). The Hcit concentrations in the non-IgE-mediated FA group were significantly decreased compared with the IgE-mediated FA group (*p* = 0.018) and the control group (*p* = 0.008). In AD children with a non-IgE-mediated FA, the Tyr levels were also significantly reduced compared with the controls (*p* = 0.009). The mean concentration of Sar was the highest in the non-IgE-mediated FA group and the lowest in the IgE-mediated FA group (*p* = 0.047). Future studies should elucidate the involvement of these AAs in the molecular pathway of IgE- and non-IgE-mediated allergic responses.

## 1. Introduction

Children with a food allergy (FA) constitute a heterogeneous group [1,2,3,4]. The causes of the symptoms, pathogenesis and clinical picture are varied [3]. Atopic dermatitis (AD) is considered one of the strongest risk factors for the development of AD in young children [5]. According to the literature data, 30% of pediatric patients suffering from severe AD also develop an FA [6]. The basis for suspecting an FA in atopic children is the medical history [1,7]. Only some of the symptoms can be assessed objectively based on physical examination. In vivo and in vitro based tests verify the immunoglobulin E (IgE)-mediated mechanism of hypersensitivity, but the correlation between them is weak. The routine diagnostic methods are characterised by a high risk of false negative and false positive results [8,9,10]. The properties of allergen extracts in in vivo and in vitro tests can be affected by prior treatment when compared with the natural starting substrate [11]. Methods for laboratory identification of a non-IgE-mediated FA are not available [8]. The gold standard for the diagnosis of an FA is a double-blind, placebo-controlled oral food challenge (OFC) test [12,13]. The OFC test has been successfully used for many years and allows for determining whether a challenged food induces symptoms that are also found in the case of a non-IgE-mediated allergy, where previously described methods cannot be used [1]. However, the availability of in vivo diagnostics in the case of children is limited and the conditions of the tests differ from the conditions of natural exposure. Sometimes allergy symptoms appear only after a few hours or a few days after eating the product, and in such cases, an elimination diet is required [14]. A diagnosis of an FA in AD children is often hampered by skin lesions, the variety of products consumed, the variability of their allergenic properties and the occurrence of allergic cross-reactivity [4].

A milestone in the history of allergy diagnostics was the discovery and evolution of component-resolved diagnostics (CRD) [15]. CRD has been applied to examine the serum concentration of IgE antibodies specific to the individual allergen molecules [16]. The first results on molecular techniques in allergology were published by Valenta et al. at the end of the 20th century [17]. Assuming that any structure that contains a protein can be an allergen, allergic reactions began to be studied not in terms of a specific food, but rather protein components. To date, scientists have discovered and described the main allergen families [4]. Knowing the specific allergenic proteins and not the source of the allergy itself brings a number of benefits [18]. Most of all, advanced CRD tools based on nanotechnology allows for distinguishing primary, true sensitisation from sensitisation due to cross-reactivity [1,18]. Furthermore, it may be useful to stratify the clinical risk associated with a particular sensitisation pattern and predict the OFC test outcome [4,15]. However, to date, the development of CRD has not solved all diagnostic problems [18]. Testing for sIgE components does not improve the risk stratification for all food products [13]. For example, in children less than 2 years of age, egg white has a higher specificity, sensitivity and positive predictive value than other egg molecules, such as ovalbumin, ovomucoid and egg yolk [19]. This strategy requires adequate interpretation to avoid unnecessarily prescribing elimination diets and adrenaline auto-injectors, which can have a negative impact on the patients’ quality of life later on [18]. Finally, the CRD applies only to type I hypersensitivity, which is associated with the release of IgE antibodies interacting with allergen epitopes [20]. Prior to the use of expensive, advanced instruments of CRD, i.e., Allergy Explorer (ALEX^2^) and Immuno Solid-Phase Allergen Chip (ISAC) assay, the mechanism of FA needs to be identified. At the moment, there is no indicator that specifically distinguishes between IgE-mediated and non-IgE-mediated FAs [8].

Currently, one of the most promising strategies for searching for new diagnostic solutions, potential biomarkers and assessing differences between people with different health status is metabolomics [21,22]. Metabolomics focuses on low-molecular-weight compounds, i.e., metabolic products such as fatty acids, nucleotides, carbohydrates and amino acids (AAs) [21]. Alterations in the human metabolome were reported to precede the appearance of clinical symptoms [23]. Two approaches are used in metabolomics studies, i.e., non-targeted and targeted analyses [21]. A targeted strategy is applied to accurately quantify a limited number of known metabolites. Using highly sensitive and selective analytical techniques, many compounds at low concentrations can be detected and quantified. A non-targeted approach allows for the classification of the studied biological samples based on a full metabolic profile. It aims to detect the structures of various metabolites specific to the metabolome, but without quantification [21,24,25].

Our aim was to determine whether the occurrence of an IgE-mediated or non-IgE-mediated FA in AD children is associated with changes in serum-free AA levels. In this study, we analysed the serum profile of 42 AAs in 76 AD children aged 2–60 months using high-performance liquid chromatography coupled with mass spectrometry (LC-MS/MS). We applied a targeted metabolomic approach. To our knowledge, this is the first paper presenting the examination of a broad panel of free AAs (both proteinogenic and non-proteinogenic) in young children with AD and FAs. The analysis of free AAs in the serum of allergic patients will contribute not only to the assessment of their diagnostic usefulness but will also help to expand the knowledge about the pathomechanisms of this condition too. AAs are key components of peptides, proteins and phospholipids [22]. Changes in AA availability are known to have a profound effect on many aspects of cellular function, e.g., gene expression, regulation of cell signaling and the transport of AAs themselves [26]. Previously, irregularities in free AA concentrations in body fluids were found in different diseases, i.e., diabetes mellitus [27,28], liver diseases [29], obesity [30,31], chronic renal failure [32,33] and neoplastic diseases [28,34,35,36].

## 2. Materials and Methods

The study was approved by the Bioethical Commission of Poznan University of Medical Sciences, Poland (decision no. 372/20), and was in accordance with the requirements of the Declaration of Helsinki. Participants were recruited from the Medical Practice of MD Joanna Matysiak in Kalisz after obtaining the written consent of their parents. The main objectives and possible benefits of the study were explained to the patients’ parents. 

### 2.1. Patients and Sample Collection

The study involved 76 children aged 2 to 60 months with chronic symptoms of AD (L20). Some patients additionally developed a severe reaction, i.e., anaphylaxis (T78.0), urticaria (L50) and/or angioedema (T78.3) following food exposure. Table 1 shows the demographic profile of the participants. The detailed characteristics of patients was presented in our previous publication [8].

Parents or legal guardians of the patients were required to carefully complete the questionnaire, which helped in the inclusion or exclusion of subjects from the study. After familiarising the parents with the nature of the study, venous blood was collected from the participants. Serum was separated via centrifugation at 300× *g* for 20 min, and finally, samples were stored at −80 °C until the appropriate analysis. 

All participants underwent a full medical examination. On the basis of the medical history, in vitro sIgE tests and an open oral food challenge (OFC) test, individuals were assigned to the appropriate groups: IgE-mediated allergy, non-IgE-mediated allergy and control group. The first group consisted of 36 AD children that suffered from IgE-mediated FAs (sIgE+; medical history+; OFC+). The non-IgE-mediated allergy group constituted 15 AD pediatric individuals with non-IgE-mediated allergy symptoms (sIgE−; medical history+; OFC+), while 25 AD patients without an FA (sIgE−; medical history+/−; OFC−) constituted the control group. Finally, we analysed changes in the serum AA profile of 76 individuals via LC-MS/MS. We used an aTRAQ kit (Sciex, Framingham, MA, USA) to quantify 42 free AAs (proteinogenic and non-proteinogenic) in the serum of participants.

### 2.2. Diagnosis of IgE-Mediated Food Allergy

We determined the concentrations of total IgE and the IgEs specific to 295 allergens (117 extracts and 178 molecular allergens) in the serum of all participants using Allergy Explorer ALEX2 (MacroArray Diagnostics, Wien, Austria). ELISA-based in vitro multiplex analysis was performed according to the manufacturer’s instructions, as previously described [8]. The results of the allergen-specific IgE in vitro tests are presented in Table S1.

Positive results of the sIgE in vivo tests were corroborated using the OFC test. Participants were selectively subjected to the OFC test based on clinical history and physical examination. Patients with detectable high concentrations of IgE specific to the suspect food and a history of anaphylaxis were not tested using the OFC test. 

### 2.3. Diagnosis of Non-IgE-Mediated Food Allergy

Patients were diagnosed with non-IgE-mediated FA using an open OFC test and observation of allergic symptoms after ingestion of the suspect food [37,38,39]. According to the procedure, suspected allergens were administered orally [14,40]. Children who underwent the OFC test did not take any medications that could affect their safety or assessment.

At the first visit, a clinical history was obtained, a detailed physical examination was performed, and parents were informed about the risks and benefits of the OFC test. Afterwards, the suspected food allergen, e.g., peanut, was fully excluded from the children’s diet for 5 weeks. The patients’ parents were then carefully instructed on how to reintroduce previously excluded products into their diet. According to the doctor’s instructions, the suspected food allergen was administered daily, gradually increasing the doses. The extension of the children’s diet was persisted until allergy symptoms renewed or the suspected food product was fully introduced. At the next follow-up visit, a detailed medical interview and physical examination of the participants were repeated. 

The patients that qualified for the study group were the children diagnosed with AD. In patients with negative sIgE results, a non-IgE-mediated FA was diagnosed if the following conditions were met: a clinical history indicating delayed-type FA with a positive OFC result with a suspicious food-allergen, during which the appearance of clinical symptoms were observed in a time typical for delayed reactions (usually 48–72 h). We assessed all clinical symptoms; however, due to the diagnosis of AD in our subjects, we especially observed the appearance and/or exacerbation of skin lesions in the form of atopic eczema. Chronic gastrointestinal symptoms were observed in only a few patients as additional symptoms and these were late reactions. Only after analysing the clinical history of each patient, the result of additional tests, namely, sIgE and the course of OFC (characteristics of the reaction, time of onset of symptoms), the diagnosis of non-IgE-mediated FA was made.

### 2.4. Sample Preparation for LC-MS/MS

First, 10 µL of 10% sulfosalicylic acid was added to a 40 µL serum sample. Then, specimens were mixed and centrifuged under appropriate conditions, i.e., 2 min at 10,000× *g*. A total of 10 µL of the supernatant was transferred to a clean Eppendorf tube and 40 µL of borate buffer (pH = 8.5) was added. After vortexing and centrifugation, 10 µL of the obtained mixture was labeled with 5 µL of pre-diluted reagent solution (aTRAQ Reagent Δ8). After the mixing and centrifugation step, samples were incubated for 30 min at 23 °C. In order to stop the labeling reaction, 5 mL of hydroxylamine was added, and samples were incubated for 15 min at room temperature. In the next step, 32 µL of the internal standard solution was transferred to a tube and the mixture was centrifuged. Evaporation in a vacuum concentrator (miVac Duo Concentrator, Genevac, Stone Ridge, NY, USA) for 15 min reduced the sample volume. Finally, 20 µL of water was added to concentrated samples and mixtures were transferred to an autosampler vial. 

### 2.5. LC-MS/MS

We measured serum-free AA concentrations using liquid chromatography–tandem mass spectrometry (LC-MS/MS), as previously described [22]. 

Measurements were carried out using a 3500 triple quad mass spectrometer (AB Sciex, Framingham, MA, USA) coupled with a liquid chromatograph. The mass spectrometer was equipped with an electrospray ionisation (ESI) source. The AAs were separated on C18 column (parameters: 4.6 mm × 150 mm, 5 µm) (Sciex, Framingham, MA, USA) with a flow rate of 0.8 mL/min using a binary gradient of water (mobile phase A) and methanol (mobile phase B), both containing 0.01% heptafluorobutyric acid and 0.1% formic acid. The gradient elution proceeded in the following way: 0 min, 2% B; 0–6 min linearly from 2 to 40% B; 6–10 min, 40% B; 10–11 min linearly from 40 to 90% B; 11–12 min, 90% B; 12–13 min linearly from 90 to 2% B; 13–18 min, 2% B. The temperature of the separation was 50 °C. The measurements were carried out in positive ionisation mode. The MS parameters were set as follows: ion spray voltage, 1500 V; entrance potential, 10 V; declustering potential, 60 V; collision energy, 30 V (except for L-lysine, argininosuccinic acid, L-homocystine, cystathionine, L-ornithine, L-cystine and δ-hydroxylysine, 50 V) and collision cell exit potential, 10 V. The temperature of the ion source was set at 600 °C, and the pressures of gas 1 and gas 2 were set to 50 psig and 50 psig, respectively. The instrument was operated in multiple reaction monitoring (MRM) mode. A full list of MRM transitions for the 42 AAs and their corresponding internal standards was provided in our previous article [21].

### 2.6. Data Analysis

Statistica 13.0 (StatSoft Inc., Tulsa, OK, USA) and MedCalc software (MedCalc Software Ltd., Ostend, Belgium) were used for the data analysis. We applied univariate statistical tests. In most analyses, *p* < 0.05 was statistically significant. The normality of the data distribution was checked using the Shapiro–Wilk test. To compare the data between the studied groups, one-way ANOVA with the least significant difference (LSD) post hoc test was applied. For each differentiating factor, a standard univariate receiver operating characteristic (ROC) curve was performed using the MedCalc software. The ROC curves provide an assessment of the specificity and sensitivity of the discriminant factor. The area under the curve (AUC) was calculated using DeLong’s method. A greater AUC means a better classification of subjects into one of the groups [21,22].

## 3. Results

The employed LC-MS/MS method enables the determination of 42 AAs (proteinogenic and non-proteinogenic) in a single run. We detected and quantified 34 out of 42 AAs in the analysed samples (Table 2). The remaining eight AAs occurred below the lower quantification level in most of analysed samples. The following AAs were not detected and quantified in our study: O-phosphoethanolamine (PEtN), L-homocystine (Hcy), argininosuccinic acid (Asa), L-anserine (Ans), L-carnosine (Car), O-phospho-L-serine (PSer), cystathionine (Cth) and L-cystine (Cys). Concentrations of the quantified AAs in the analysed samples are presented in Table 2. Table 2 also contains the limit of quantitation (LOQ) of the method for all AAs (≤1 µM). The linear dynamic range was from the LOQ value to ≥10,000 mM.

**Figure 1:**
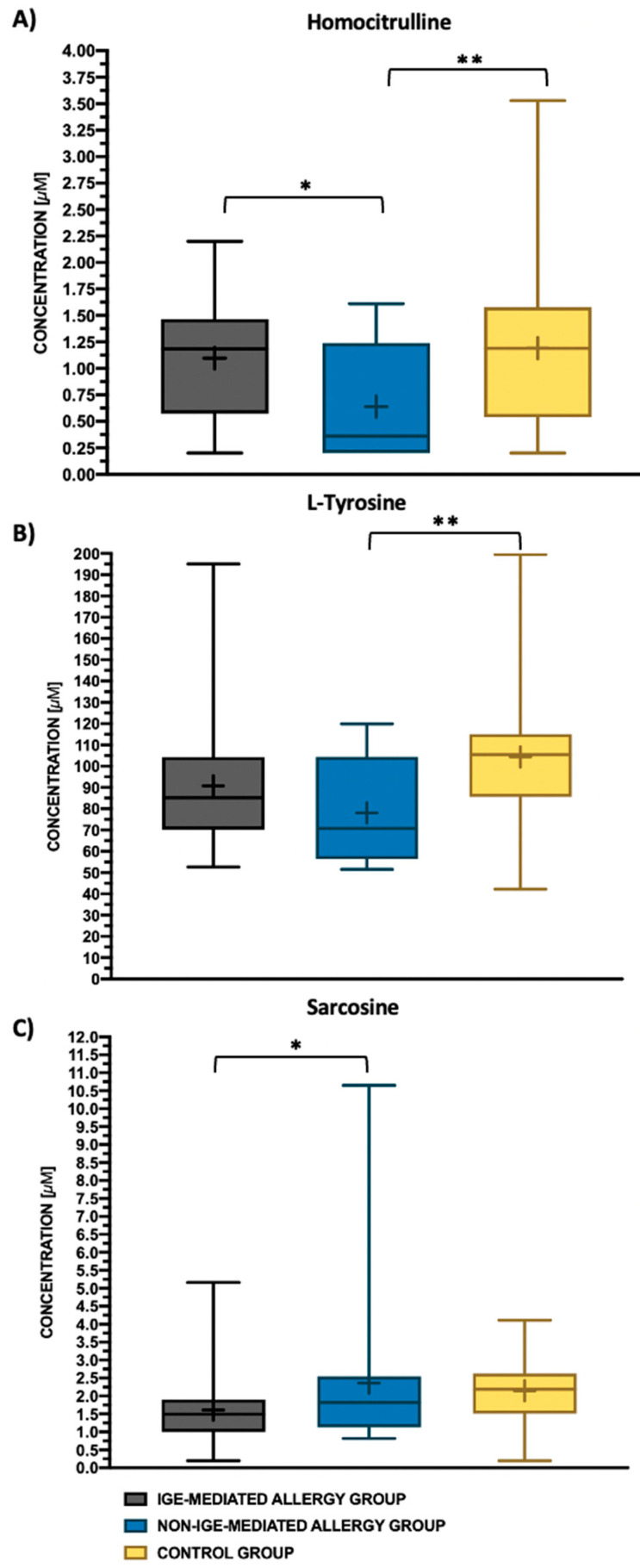
Box and whisker plot for the three amino acids—homocitrulline (**A**), L-tyrosine (**B**), sarcosine (**C**)—used to differentiate the studied groups: IgE-mediated allergy group (grey box), non-IgE-mediated allergy group (blue box) and control group (yellow box). Top and bottom whiskers in the figure represent the maximum and minimum amino acid concentration values, the line inside each box shows the median value and the plus represents the mean value. The upper and lower borders of the boxes represent the 75th and 25th percentile values, respectively. Least significant difference (LSD) post hoc test: ** *p*-value < 0.01, * *p*-value < 0.05.

To evaluate differences in the serum AA concentrations between three groups of AD children, one-way ANOVA with the LSD post hoc test was used. Univariate analysis showed that the average concentration of homocitrulline (Hcit; *p* = 0.026), sarcosine (Sar; *p* = 0.024) and L-tyrosine (Tyr; *p* = 0.018) differed in the IgE-mediated allergy group, the non-IgE-mediated allergy group and the control group (Table 3 and Table S2). The average concentration of Hcit in the non-IgE-mediated allergy patients was significantly decreased compared with the IgE-mediated allergy group (LSD post hoc test; *p* = 0.018) and the control group (LSD post hoc test; *p* = 0.008) (Figure 1A). In children with a non-IgE-mediated FA, the level of Tyr was also significantly reduced compared with the control group (LSD post hoc test; *p* = 0.009), (Figure 1B). The average value of Sar concentration was the highest in AD children with a non-IgE-mediated FA and the lowest in AD children with an IgE-mediated FA. The difference between the IgE-mediated and non-IgE-mediated allergy patients was statistically significant (LSD post hoc test; *p* = 0.047) (Figure 1C).

**Figure 2:**
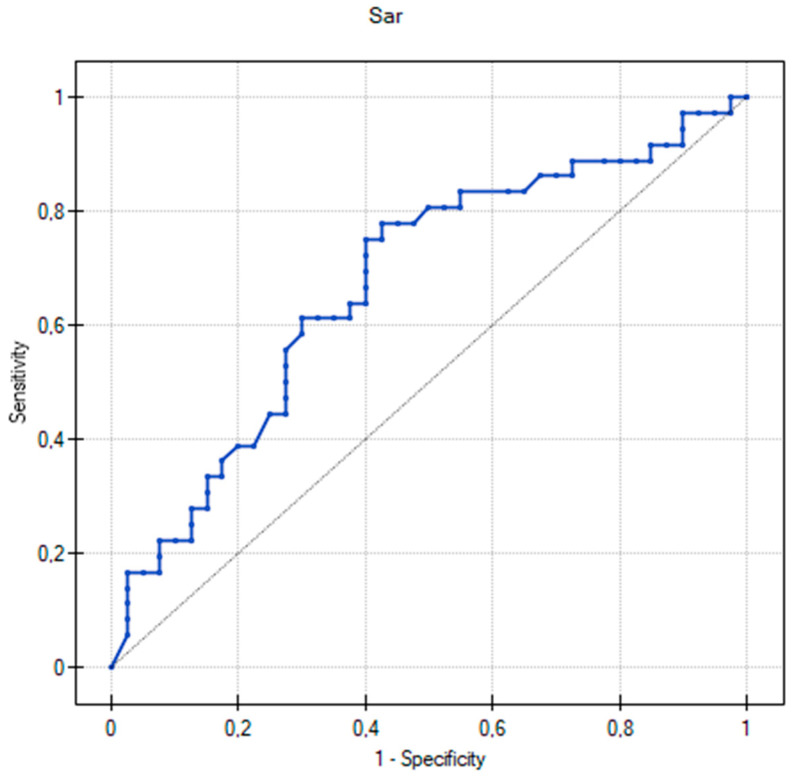
Univariate receiver operating characteristic (ROC) curve for sarcosine (Sar) predicting an IgE-mediated food allergy in children with atopic dermatitis.

**Figure 3:**
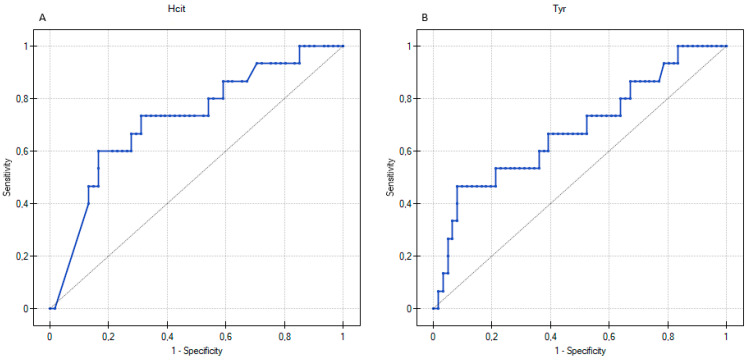
Univariate receiver operating characteristic (ROC) curves for homocitrulline (Hcit (**A**)) and L-tyrosine (Tyr (**B**)) predicting a non-IgE-mediated food allergy in children with atopic dermatitis.

ROC curves were calculated to determine a cut-off value of differentiating AAs for AD children with different mechanisms of an FA and patients without an FA (Table 4). In this study, an AUC of approximately 0.7 was satisfactory. The ROC curve for Sar demonstrated that the concentration value of 0.35 µM discriminated AD children with an IgE-mediated FA and without an IgE-mediated FA with the specificity of 60% and sensitivity of 75% (Figure 2, Table 4). The AUC was 0.67, and considering the confidence interval (CI) of 95%, it ranged from 0.549 to 0.796. In turn, Hcit and Tyr may be considered as potential indicators that differentiated non-IgE-mediated FA in AD patients aged 2–60 months (Table 4). The highest AUC value (0.73) was reported for Hcit, with a specificity of 69% and a sensitivity of 73% at a cut-off value of 0.422 µM (Figure 3A). The cut-off value calculated from the ROC curve for Tyr was 0.32 µM (sensitivity = 53%, specificity = 80%, AUC = 0.68, 95% CI = 0.517–0.844) (Figure 3B).

## 4. Discussion

In this study, we successfully analysed the serum concentrations of 34 free amino acids (AAs) using a triple quadrupole mass spectrometer coupled with a liquid chromatography instrument. Serum samples were collected from 76 pediatric patients aged 2–60 months with atopic dermatitis (AD). We compared the AA profiles of AD children diagnosed with an IgE-mediated food allergy (FA) and a non-IgE-mediated FA, as well as AD pediatric patients without FA. The applied methodology ensured the high sensitivity and specificity of measurements and enabled parallel quantification of a wide AA profile in a small sample volume. The benefits of using aTRAQ reagents with the LC-MS/MS apparatus are supported by literature data [41,42]. The use of aTRAQ reagents for the quantitative analysis of AA in biological fluids was shown to increase the specificity of the method and significantly reduce the analysis time (one analysis cycle lasts 18 min) [22]. Using LC-MS/MS instrumentation and aTRAQ kit, we previously examined alterations in serum-free AA profiles in children aged 3–18 years suffering from allergic asthma. The results showed decreased levels of L-valine, taurine and DL-β-aminoisobutyric acid and increased serum concentrations of ƴ-amino-n-butyric acid and L-arginine in pediatric patients suffering from asthma when compared with children without asthma and allergy [21]. Morris et al. studied a population of patients range of 2 to 52 years (mean age 12–14 years; median age 10 years) and showed a significantly reduced concentration of arginine in the serum of participants with allergic asthma [43]. In the present study, we identified homocitrulline (Hcit), sarcosine (Sar) and L-tyrosine (Tyr) as features that differentiated AD patients aged 2–60 months with an IgE-mediated FA, with a non-IgE-mediated FA and without a FA. The altered concentrations of these AAs suggest their roles in the pathogenesis of allergic reaction to food in atopic children. This is the first AA report on the population of pediatric patients with chronic symptoms of AD and FA. Studies conducted in pediatric populations require specific evaluation. The immune system of young children is often immature. In accordance with the literature, the human immune system matures over several years [44]. In patients with chronic AD aged 0–5 years, we already found changes in the profile of inflammatory cytokines depending on the presence of FA and the pathway of the allergic response [8]. These results have not yet been demonstrated in the adult patient population. Changes in serum-free AAs may also vary with the age of patients and be directly related to the immune system and its maturation [21,45,46].

**Figure 4:**
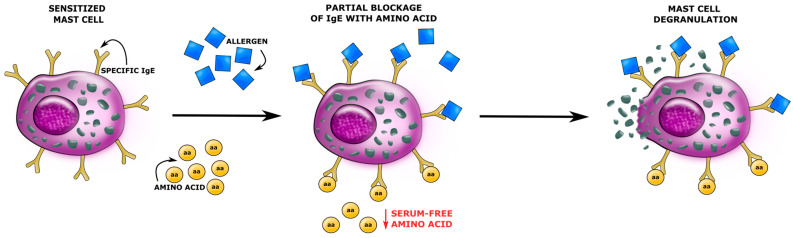
Amino acids bind to IgE-sensitised mast cells (created with Inkscape 1.2.2).

The first AA that differentiated the population of AD children was Sar, which is an N-methyl derivative of glycine. It is found in muscles and other tissues [47]. Sar can be metabolised to glycine by sarcosine dehydrogenase, while glycine-N-methyl transferase generates sarcosine from glycine [48]. Sar naturally occurs in the metabolism of choline to glycine. Sar was identified as a biomarker for invasive prostate cancer [49]. Its level increases significantly during prostate cancer progression to metastasis and can be detected in urine. Sar can also act as a biomarker of eosinophilic esophagitis (EoE), which is an allergic inflammatory condition of the esophagus that involves eosinophils [50]. This condition is not well understood, but a significant role is attributed to the allergic response. EoE is thought to be an allergic reaction to ingested food [51]. It is estimated that 10–20% of EoE pediatric patients also have symptoms of immediate IgE-mediated FA [52]. According to our results, the serum concentration of Sar was significantly decreased in AD children with the IgE-mediated FA when compared with AD pediatric patients suffering from the non-IgE-mediated FA. S. Chung et al. studied the effect of AAs on IgE binding to peanut allergens. Researchers reported that some AAs can inhibit IgE binding to food allergens [53,54]. Consistent with these assumptions, AD children with a high IgE-mediated allergic response may have lower detectable level of serum-free Sar compared with other AD pediatric patients (Figure 4). 

Hcit and Tyr may be considered as potential indicators of a non-IgE-mediated FA in AD patients aged 2–60 months. The concentrations of Hcit and Tyr were significantly decreased in the non-IgE-mediated allergy group in comparison with other AD children. Hcit belongs to the class of organic compounds known as l-alpha-amino acids, which have the L-configuration of the alpha-carbon atom [55]. It is a secondary metabolite that can act as a defense or signaling molecule. Previously, A. Desmons et al. quantified Hcit in the serum of patients aged more than 50 years with chronic kidney disease using LC-MS/MS [56,57]. According to their results, serum Hcit concentrations were increased in adult individuals with chronic renal failure [57]. Hcit was also shown to be associated with EoE in pediatric patients. In children aged 1–13 years suffering from EoE, Hcit was significantly reduced in the urine compared with healthy children. Tyr is an essential amino acid that easily crosses the blood–brain barrier [58]. In addition to its role as a precursor for neurotransmitters, Tyr plays an important role for the function of many proteins [59]. Tyr can be phosphorylated by specialised protein kinases [60]. Tyr phosphorylation is major step in the regulation of enzymatic activity and signal transduction. According to literature data, Lck/yes-related protein tyrosine kinase (Lyn) is involved in the pathogenesis of inflammation and allergic disease [61]. The interaction between Lyn and FcεRIβ is essential for mast cell activation and regulates the allergic response [62]. Tyr phosphorylation of the FcεRI β subunit by Lyn kinase plays a key role in this signaling cascade [63]. Research shows that Lyn can exhibit both positive and negative regulatory effects on the signaling pathway [61]. Odom et al. showed that Lyn-deficient mice are susceptible to specific allergic diseases [64], while according to Wang et al. and Zhu and Bertics, Lyn is associated with a high severity of allergic asthma in mouse models and is a key molecule of highly expressed blood eosinophils [65,66]. Wypych et al. demonstrated that the microbial metabolism of Tyr protects against allergic inflammation of the airways [67]. In addition, a number of clinical studies showed that Tyr can be used as an adjuvant in allergy vaccines [68]. The adsorption of allergenic molecules to Tyr enhanced the induction of IgG antibodies, but did not stimulate the IgE antibody or delayed hypersensitivity [69]. Decreased levels of Tyr and Hcit in the serum of AD children with non-IgE-mediated FA suggest their role in the pathogenesis of allergic reaction to food, regardless of IgE production.

The identified abnormalities in the AA profile are the basis for more advanced research and additional explanation and differentiation of the molecular mechanisms of FAs in AD children. At the moment, there is no accurate, specific indicator that differentiates IgE-mediated and non-IgE-mediated allergic responses [70]. Mast cells are crucial immune effector cells in all allergic reactions and undergo activation by both IgE-mediated and non-IgE-mediated mechanisms [71]. Activation and degranulation of mast cells mediated by the immunoglobulin E receptor (FcεRI) are well-described and understood mechanisms of allergy and hypersensitivity reactions [72,73]. In addition to FcεR1, mast cells express other receptors activated through the non-IgE-mediated pathway [74]. However, non-IgE-mediated mast cell activation is less studied and not well recognised. Non-IgE-mediated hypersensitivity reactions do not elicit a classic antigen-specific immune response, but they do elicit histamine and cytokine release, complement system activation and atypical eicosanoid synthesis [75]. We can speculate that observed changes in the serum-free AA profile in AD children may be associated with mast cell metabolism, which varies between IgE- and non-IgE-mediated degranulation [71].

This is the first study that used aTRAQ reagents with the LC-MS/MS system to determine the concentrations of serum-free AAs in AD children with FAs. Our study was novel, and it was impossible to precisely compare the outcomes for the studied population with other researchers. Since the number of analysed samples was limited, this study had a pilot nature. However, our data provide an interesting and fresh insight into the molecular basis of the allergic reaction in AD children. Changes in the metabolome may be prognostic. According to the literature, they appear before allergen-specific antibodies [8]. Early diagnosis of FA is particularly relevant in pediatric patients with severe AD. Identification and elimination of the allergenic factor can improve the course and efficacy of treatment. In the future, the determination of changes in the AA profile in serum may be a minimally invasive diagnostic tool in allergic diseases in pediatric patients. However, more advanced, large-scale studies are required to confirm the diagnostic value of AAs in predicting FAs in children with AD. Defining the actual sensitivity and specificity of identified AAs will be possible thanks to the punctual comparison with sIgE. 

## 5. Conclusions

Amino acids (AAs) can act as mediators of immune system activity. Changes in the serum-free AA profile may be associated with an allergic reaction and depend on its molecular pathway. In this study, we identified homocitrulline (Hcit), sarcosine (Sar) and L-tyrosine (Tyr) as differentiating features between atopic dermatitis (AD) children with and without food allergies (FAs). Future studies should investigate and elucidate the involvement of these AAs in the molecular pathway of IgE- and non-IgE-mediated allergic responses.

## Figures and Tables

**Table 1:** Characteristics of participants.

Characteristics of Participants	IgE-Mediated Food Allergy	Non-IgE-Mediated Food Allergy	Control Group Lack of Food Allergy
No. of subjects	36	15	25
Sex			
Male	19	11	14
Female	17	4	11
Age (months)			
Median	23.5	12	20.5
Mean	25.81	13.2	26.6
Range	6–60	6–36	2–60
Eczema (for the last 1.5 months or more)	36	15	25
Patient age on the onset of eczema (months)			
Median	3	3	4
Mean	4.4	3.73	8.9
Range	1–24	1–10	2–36
Atopic dermatitis (L20)	36	15	25
Allergic urticaria (L50)	8	0	2
Angiodema (T78.3)	5	0	0
Anaphylactic shock (T78.0)	5	0	0
Chronic symptoms of the digestive system	Abdominal pain, abdominal gas, diarrhea, Constipation, mucus in the stool	Colic, abdominal pain, abdominal gas, vomiting, downpouring, diarrhea, constipation, mucus in the stool	Diarrhea
Early childhood asthma	5	2	1
Allergic rhinitis	13	4	3

**Table 2:** Concentrations of 34 amino acids determined in the serum samples. Concentration values are given in µM. SD—standard deviation, LOQ—limit of quantification.

Amino Acid	Abbreviation	LOQ (µM)	IgE-Mediated Allergy Group			Non-IgE-Mediated Allergy Group			Control Group		
			Median	Mean	SD	Median	Mean	SD	Median	Mean	SD
1-Methyl-L-histidine	1MHis	0.2	9.46	13.5	11.61	12.26	14.00	6.10	12.04	18.36	14.62
3-Methyl-L-histidine	3MHis	0.2	3.18	6.02	10.05	3.25	5.60	5.64	3.82	9.18	11.83
L-α-Aminoadipic acid	Aad	0.2	1.49	1.56	0.79	1.89	1.94	0.67	1.50	1.83	1.16
L-α-Amino-n-butyric acid	Abu	0.5	23.24	23.96	10.58	26.24	28.41	12.54	27.93	26.87	11.26
L-Alanine	Ala	0.2	487.33	497.88	120.79	476.24	484.16	67.99	497.42	507.48	116.11
L-Arginine	Arg	0.5	157.53	154.46	45.08	136.8	153.96	73.77	155.37	162.25	53.65
L-Asparagine	Asn	0.5	86.22	101.36	38.85	88.53	91.12	23.65	101.24	101.98	35.99
L-Aspartic acid	Asp	0.1	40.36	40.82	13.01	34.26	33.43	9.54	31.55	34.53	10.39
D,L-β-Aminoisobutyric acid	bAib	0.2	2.60	3.23	2.14	2.24	2.80	1.48	3.00	3.16	1.26
β-Alanine	bAla	0.5	13.85	21.16	28.11	14.97	16.38	5.96	15.42	19.41	9.10
L-Citrulline	Cit	0.5	26.31	25.30	8.18	23.82	25.68	10.12	27.17	28.24	6.69
Ethanolamine	EtN	0.5	12.67	13.38	3.92	13.04	13.70	3.51	11.34	11.55	2.73
ƴ-Amino-n-butyric acid	GABA	0.05	5.45	5.56	3.75	0.79	4.00	5.33	5.40	4.43	2.85
L-Glutamine	Gln	0.5	823.58	819.94	171.82	735.97	770.05	133.73	836.35	803.581	117.63
L-Glutamic acid	Glu	0.5	112.96	117.53	43.51	123.11	133.74	33.99	109.08	110.42	37.23
Glycine	Gly	1	277.38	276.23	57.56	265.30	272.11	45.32	277.75	279.73	63.14
Homocitrulline	Hcit	0.2	1.19	1.1	0.53	0.36	0.64	0.53	1.19	1.19	0.77
L-Histidine	His	0.5	96.55	106.09	40.76	94.54	94.33	17.55	105.72	103.56	16.48
Hydroxylysine	Hyl	0.5	0.96	1.15	0.70	1.09	1.13	0.46	0.92	1.29	1.18
Hydroxy-L-proline	Hyp	0.2	25.45	26.40	10.04	25.62	26.69	7.39	22.19	24.15	9.52
L-Isoleucine	Ile	0.5	102.42	110.53	42.02	127.557	118.11	41.52	122.50	125.71	40.37
L-Leucine	Leu	0.5	198.07	199.62	59.31	214.98	210.65	66.75	220.49	216.18	65.80
L-Lysine	Lys	0.5	208.09	218.25	70.54	234.984	217.26	53.03	235.83	224.13	61.29
L-Methionine	Met	0.1	34.51	37.48	16.79	31.86	37.72	15.53	44.99	44.68	13.73
L-Ornithine	Orn	0.5	114.21	119.10	35.27	102.20	118.19	39.01	124.23	123.74	32.020
L-Phenylanalanine	Phe	0.2	91.55	99.06	27.31	83.36	93.78	35.79	101.94	101.26	22.16
L-Proline	Pro	0.1	246.17	252.72	69.94	227.47	235.48	89.611	259.45	287.29	110.31
Sarcosine	Sar	0.2	1.49	1.61	0.96	1.82	2.36	2.4	2.19	2.15	0.91
L-Serine	Ser	0.5	250.01	252.10	52.2	237.09	235.39	36.63	238.32	245.41	47.09
Taurine	Tau	0.5	141.24	152.48	41.63	164.857	160.52	37.57	145.79	140.60	37.55
L-Threonine	Thr	0.2	134.50	150.37	55.3	175.23	166.32	46.35	157.40	157.43	44.36
L-Tryptophan	Trp	0.1	56.80	63.70	18.40	69.25	68.24	13.39	64.28	66.37	15.30
L-Tyrosine	Tyr	0.5	85.24	90.78	29.16	70.75	78.09	23.87	105.38	104.34	34.49
L-Valine	Val	0.2	285.72	293.78	88.58	289.63	307.93	92.19	308.02	327.97	86.57

**Table 3:** Results of the one-way ANOVA test. *p*-value—probability value.

Amino Acid	Abbreviation	*p*-Value	
		ANOVA Kruskal–Wallis Test	ANOVA Test
1-Methyl-L-histidine	1MHis	0.160	
3-Methyl-L-histidine	3MHis	0.159	
L-α-Aminoadipic acid	Aad	0.125	
L-α-Amino-n-butyric acid	Abu	0.503	
L-Alanine	Ala		0.885
L-Arginine	Arg	0.615	
L-Asparagine	Asn	0.746	
L-Aspartic acid	Asp		0.108
D,L-β-Aminoisobutyric acid	bAib	0.564	
β-Alanine	bAla	0.417	
L-Citrulline	Cit	0.232	
Ethanolamine	EtN	0.061	
ƴ-Amino-n-butyric acid	GABA	0.232	
L-Glutamine	Gln		0.534
L-Glutamic acid	Glu		0.156
Glycine	Gly	0.967	
Homocitrulline	Hcit	0.026	
L-Histidine	His	0.303	
Hydroxylysine	Hyl	0.647	
Hydroxy-L-proline	Hyp	0.331	
L-Isoleucine	Ile	0.262	
L-Leucine	Leu	0.565	
L-Lysine	Lys	0.727	
L-Methionine	Met	0.05	
L-Ornithine	Orn	0.634	
L-Phenylanalanine	Phe	0.402	
L-Proline	Pro		0.277
Sarcosine	Sar	0.024	
L-Serine	Ser		0.618
Taurine	Tau	0.200	
L-Threonine	Thr	0.326	
L-Tryptophan	Trp	0.330	
L-Tyrosine	Tyr	0.018	
L-Valine	Val	0.246	

**Table 4:** Utility of three differentiating amino acids (sarcosine, homocitrulline, L-tyrosine) in predicting IgE-mediated and non-IgE-mediated food allergies in children with atopic dermatitis. Statistical significance *p* < 0.05. *p*-value—probability value, AUC—area under the curve.

Amino Acid	IgE-Mediated Food Allergy		Non-IgE-Mediated Food Allergy	
	AUC (95%CI)	*p*-Value	AUC (95%CI)	*p*-Value
Sarcosine	0.67	0.01	0.53	0.73
Homocitrulline	0.58	0.26	0.73	0.01
L-tyrosine	0.54	0.53	0.68	0.03

## Data Availability

The data presented in this study are available in the Supplementary Materials.

## Supplementary Materials

The following supporting information can be downloaded at: https://www.mdpi.com/article/10.3390/biomedicines11071919/s1: Table S1. Results of sIgE in vitro tests; Table S2. Results of Shapiro–Wilk test.
