# Supplementary material for: Amino Acid Profiling Identifies Disease-Specific Signatures in IgE-Mediated and Non-IgE-Mediated Food Allergy in Pediatric Patients with Atopic Dermatitis
Source: Biomedicines. 2023 Jul 6;11(7):1919. doi: 10.3390/biomedicines11071919 (PMC10377369; doi:10.3390/biomedicines11071919)
Supplement: Supplementary file 1 [file biomedicines-11-01919-s001.zip › biomedicines-2458503-supplementary.pdf]

Table S1. Results of sIgE *in vitro* tests.

ALLERGEN	SOURCE	E/M	PROTEIN FAMILY	TOTAL STUDY POPULATION			ALLERGIC PATIENTS			
				Mean	SD	Median	%	Mean	SD	Median
Sec c	Rye	E		0.273	0.268	0.1	34.2	0.585	0.244	0.53
Pop n	Black poplar	E		0.381	0.524	0.13	34.2	0.918	0.633	0.8
Che q	Quinoa	E		0.226	0.201	0.1	23.7	0.538	0.186	0.51
Cup s	Cypress (evergreen)	E		0.195	0.186	0.1	22.4	0.483	0.213	0.4
Sol spp.	Fire ant	E		0.174	0.121	0.1	19.7	0.391	0.09	0.36
nAra h 1	Peanut	M	Globulin 7/8S	2.104	7.669	0.1	18.4	10.93	14.959	3.165
rBet v 1	Silver birch	M	PR-10	2.004	6.837	0.1	17.1	11.175	13.108	4.4
nAra h 2	Peanut	M	Albumin 2S	2.553	8.335	0.1	17.1	14.434	15.359	9.81
rPhl p 1	Meadow timothy	M	Beta-expansin	1.628	5.907	0.1	15.8	8.981	11.779	4.22
rCry j 1	Japanese cryptomeria	M	Pectin lyase	0.241	0.530	0.1	15.8	0.896	1.122	0.46
nAra h 6	Peanut	M	Albumin 2S	2.376	8.052	0.1	15.8	14.513	15.353	7.975
Pha v	White beans	E		0.181	0.157	0.1	15.8	0.498	0.16	0.465
Gal d	Egg white	E		0.522	1.838	0.1	15.8	2.718	3.956	0.915
rCan f 1	Dog	M	Lipocalin	1.058	3.694	0.1	15.8	6.143	7.465	3.385
nGal d 2	Egg white	M	Ovalbumin	0.495	1.863	0.1	14.5	2.822	4.202	0.74
rFel d 1	Cat	M	Secretoglobin	1.834	6.492	0.1	14.5	12.05	13.006	3.35
nLol p 1	Ryegrass pollen	M	Beta-expansin	0.921	3.580	0.1	13.2	5.749	7.831	2.65
nCup a 1	Cypress (Arizonian)	M	Pectin lyase	0.228	0.531	0.1	13.2	1.056	1.262	0.49
nGal d 4	Egg white	M	C-type lysozyme	0.375	0.965	0.1	13.2	2.155	1.85	1.775
rPhl p 5.0101	Meadow timothy	M	Grasses group 5/6	1.678	6.397	0.1	11.8	12.066	13.665	7.06
Pan m	Millet	E		0.165	0.141	0.1	11.8	0.504	0.163	0.55
rFag s 1	European beech	M	PR-10	1.366	5.178	0.1	11.8	10.746	11.253	8.19
Ulm c	English elm	E		0.174	0.213	0.1	11.8	0.639	0.359	0.49
nAra h 3	Peanut	M	Globulin 11S	1.160	4.820	0.1	11.8	9.038	11.216	3.59
Cuc p	Pumpkin seeds	E		0.156	0.129	0.1	11.8	0.453	0.18	0.39
rFel d 7	Cat	M	Lipocalin	0.713	2.493	0.1	11.8	5.269	5.379	4.13
Sec c	Rye pollen	E		0.580	3.234	0.1	10.5	4.142	8.597	0.8
rCyn d 1	Bermuda grass	M	Beta-expansin	0.703	3.153	0.1	10.5	5.117	7.865	2.05
Cor a	Hazel	E		0.327	0.919	0.1	10.5	2.238	1.987	1.16
Ana o	Cashew nut	E		0.425	1.502	0.1	10.5	3.148	3.626	0.725
Ave s	Oat	E		0.186	0.347	0.1	9.2	0.879	0.876	0.5
rCor a 1.0103	Hazel	M	PR-10	0.585	2.140	0.1	9.2	5.331	4.991	2.48
rCor a 1.0401	Hazelnut	M	PR-10	0.354	1.344	0.1	9.2	2.833	3.582	1.4
rMala s 6	Malassezia sympodialis	M	Cyclophilia	0.137	0.077	0.1	9.2	0.334	0.026	0.33
rPhl p 6	Meadow timothy	M	Grasses group 5/6	0.729	4.651	0.1	7.9	8.053	14.687	2.015
Cyn d	Bermuda grass	E		0.355	1.243	0.1	7.9	2.831	3.164	0.6
rAln g 1	Black alder	M	PR-10	0.507	2.439	0.1	7.9	5.212	7.161	1.765
Sol t	Potato	E		0.174	0.269	0.1	7.9	0.762	0.721	0.43
nCor a 9	Hazelnut	M	Globulin 11S	0.342	1.113	0.1	7.9	3.157	2.662	2.695
nCor a 14	Hazelnut	M	Albumin 2S	0.315	0.926	0.1	7.9	2.767	2.081	2.21
Car i	Pecan	E		0.467	2.247	0.1	7.9	4.673	6.687	0.9
Pru du	Almond	E		0.234	0.621	0.1	7.9	1.763	1.53	1.235
Tri fo	Fenugreek seeds	E		1.195	6.072	0.1	7.9	13.943	17.045	3.36
Cic a	Chickpea	E		0.528	2.824	0.1	7.9	5.445	8.648	1.305
rFra a 1+3	Strawberry	M	PR-10 + LTP	0.598	3.051	0.1	7.9	6.377	9.037	2.855
nGal d 1	Egg white	M	Ovomucoid	0.432	1.544	0.1	7.9	4.28	3.755	3.7
rAlt a 1	Alternaria alternata	M	Group Alt a 1	1.789	7.454	0.1	7.9	21.452	16.852	17.92
Fag e	Buckwheat	E		0.186	0.295	0.1	6.6	1.026	0.732	0.57

All s	Garlic	E		0.211	0.449	0.1	6.6	1.776	0.669	1.69
Dau c	Carrot	E		0.159	0.328	0.1	6.6	0.96	0.974	0.62
nCor a 11	Hazelnut	M	Globulin 7/8S	0.210	0.413	0.1	6.6	1.592	0.727	1.6
nJug r 4	Walnut	M	Globulin 11S	0.206	0.516	0.1	6.6	1.662	1.332	1.47
Ses i	Sesame	E		0.167	0.293	0.1	6.6	1.092	0.623	0.92
nSes i 1	Sesame	M	Albumin 2S	0.202	0.443	0.1	6.6	1.56	0.997	1.19
Hel a	Sunflower seeds	E		0.281	1.121	0.1	6.6	2.538	3.688	0.49
Pap s	Poppy seeds	E		0.285	0.936	0.1	6.6	2.85	2.506	1.32
nAct d 2	Kiwi	M	TLP	0.146	0.080	0.1	6.6	0.364	0.076	0.34
rMal d 1	Apple	M	PR-10	0.712	2.928	0.1	6.6	9.33	7.126	10.67
nBos d 4	Cow milk	M	α-lactalbumin	0.384	1.732	0.1	6.6	4.392	5.329	2.17
nBos d 8	Cow milk	M	Casein	0.176	0.340	0.1	6.6	1.218	0.77	1.02
Ovi a	Sheep milk	E		0.137	0.206	0.1	6.6	0.856	0.526	0.6
nGal d 3	Egg white	M	Ovotransferrin	0.248	0.957	0.1	6.6	2.27	3.086	0.93
rCan f 4	Dog	M	Lipocalin	1.091	5.033	0.1	6.6	15.062	13.271	17.46
rCan f 6	Dog	M	Lipocalin	0.634	3.954	0.1	6.6	8.174	13.297	2.25
rHom s LF	Human lactoferrin homolog	M	CCD	0.131	0.130	0.1	6.6	0.564	0.234	0.44
rPhl p 2	Meadow timothy	M	Expansin	0.217	0.619	0.1	5.3	2.273	1.675	2.065
Art v	Mugwort	E		0.394	2.107	0.1	5.3	5.513	7.528	1.6
rArt v 1	Mugwort	M	Plant defensin	0.344	1.623	0.1	5.3	4.688	5.486	2.1
nPla a 2	Maple-leaved plane tree	M	Polygalacturonase	0.175	0.399	0.1	5.3	1.438	1.151	1.07
rDer f 1	Dermatophagoides farinae	M	Cysteine protease	0.760	3.888	0.1	5.3	12.64	11.756	11.38
rDer p 1	Dermatophagoides pteronyssinus	M	Cysteine protease	0.665	3.637	0.1	5.3	10.835	11.923	6.32
nJug r 6	Walnut	M	Globulin 7/8S	0.146	0.189	0.1	5.3	0.838	0.399	0.705
rGly m 6	Soy	M	Globulin 11S	0.391	1.652	0.1	5.3	5.623	4.792	4.455
Cap h	Goat milk	E		0.160	0.308	0.1	5.3	1.218	0.788	0.96
Sal k	Prickly brine	E		0.120	0.058	0.1	3.9	0.373	0.025	0.38
Sin a	Mustard	E		0.171	0.399	0.1	3.9	1.89	0.974	1.4
nSin a 1	Mustard	M	Albumin 2S	0.298	1.193	0.1	3.9	5.047	3.549	3.65
Cap a	Paprika	E		0.120	0.058	0.1	3.9	0.36	0.028	0.38
Jug r	Walnut	E		0.154	0.228	0.1	3.9	1.167	0.49	1.16
Ail a	Gingerbread	E		0.129	0.068	0.1	3.9	0.383	0.034	0.37
rDer p 23	Dermatophagoides pteronyssinus	M	Class III chitinase (perithophin protein domain)	0.631	3.820	0.1	3.9	13.487	14.057	6.06
All c	Onion	E		0.121	0.068	0.1	3.9	0.42	0.124	0.37
rApi g 1	Celery	M	PR-10	0.162	0.275	0.1	3.9	1.437	0.45	1.75
rDau c 1	Carrot	M	PR-10	0.192	0.561	0.1	3.9	2.39	1.71	1.45
nJug r 1	Walnut	M	Albumin 2S	0.425	2.514	0.1	3.9	8.287	9.787	2.38
nJug r 2	Walnut	M	Globulin 7/8S	0.187	0.474	0.1	3.9	2.223	1.169	2.43
rAna o 3	Cashew nut	M	Albumin 2S	0.141	0.283	0.1	3.9	1.127	1.011	0.53
Ber e	Brazil nut	E		0.283	0.873	0.1	3.9	4.573	0.336	4.77
Lup a	Lupine seeds	E		0.593	3.328	0.1	3.9	12.413	11.626	4.54
nAct d 1	Kiwi	M	Cysteine protease	0.177	0.472	0.1	3.9	2.027	1.443	1.93
Bos d	Cow milk	E		0.200	0.482	0.1	3.9	2.57	0.201	2.47
nBos d 5	Cow milk	M	β-lactoglobulin	0.227	0.868	0.1	3.9	3.05	3.281	0.77
Ach d	House cricket	E		0.502	3.342	0.1	3.9	10.203	13.597	0.82
Ten m	Mealworm of millers	E		0.473	2.942	0.1	3.9	9.443	11.637	1.38
Loc m	Migratory locust	E		0.534	3.592	0.1	3.9	11.07	14.534	1.14
rAsp f 3	Aspergillus fumigatus	M	Peroxisomal protein	0.146	0.271	0.1	3.9	1.25	0.765	0.95
rMala s 11	Malassezia sympodialis	M	Mitochondrial superoxide dismutase	0.134	0.217	0.1	3.9	0.957	0.701	0.63
Can f	Dog	E		0.303	1.389	0.1	3.9	5.173	4.92	3.15
nMus m 1	House Mouse	M	Lipocalin	0.214	0.851	0.1	3.9	2.993	3.208	0.78
rOry c 3	Rabbit epithelium	M	Secretoglobin	0.441	2.474	0.1	3.9	8.703	9.167	2.6

Pas n	Pospalum notatum	E		0.130	0.213	0.1	2.6	1.16	0.79	1.16
rMer a 1	Annual peak	M	Profilin	0.121	0.130	0.1	2.6	0.905	0.105	0.905
Ama r	Rough amaranth	E		0.122	0.069	0.1	2.6	0.465	0.015	0.465
Urt d	Stinging nettle	E		0.110	0.047	0.1	2.6	0.375	0.045	0.375
rBet v 2	Silver birch	M	Profilin	0.164	0.454	0.1	2.6	2.205	1.875	2.205
nPho d 2	Date palm	M	Profilin	0.175	0.580	0.1	2.6	2.93	2.23	2.93
Aca m	Acacia	E		0.117	0.097	0.1	2.6	0.705	0.015	0.705
Jun a	Juniper	E		0.123	0.175	0.1	2.6	0.97	0.65	0.97
rBlo t 10	Blomia tropicalis	M	Tropomyosin	0.544	3.739	0.1	2.6	16.97	15.94	16.97
rDer p 2	Dermatophagoides pteronyssinus	M	NPC2 family	0.124	0.202	0.1	2.6	1.87	0.0	1.87
rDer p 5	Dermatophagoides pteronyssinus	M	Unknown	0.115	0.095	0.1	2.6	0.67	0.16	0.67
rDer p 10	Dermatophagoides pteronyssinus	M	Tropomyosin	0.542	3.712	0.1	2.6	16.87	15.8	16.87
Sola l	Tomato	E		0.115	0.073	0.1	2.6	0.525	0.135	0.525
nAra h 8	Peanut	M	PR-10	0.172	0.549	0.1	2.6	2.71	2.2	2.71
nAra h 15	Peanut	M	Oleosin	0.119	0.065	0.1	2.6	0.475	0.015	0.475
Mac inte	Makadamia	E		0.163	0.423	0.1	2.6	2.135	1.665	2.135
nMac i	Makadamia	M	Albumin 2S	0.149	0.395	0.1	2.6	1.94	1.62	1.94
Len c	Lentils	E		0.133	0.183	0.1	2.6	1.225	0.185	1.225
Pis s	Pea	E		0.144	0.257	0.1	2.6	1.665	0.365	1.665
Car p	Papaya	E		0.112	0.064	0.1	2.6	0.47	0.15	0.47
rCuc m 2	Melon	M	Profilin	0.189	0.568	0.1	2.6	3.475	1.075	3.475
Mus a	Banana	E		0.117	0.065	0.1	2.6	0.47	0.09	0.47
Pyr c	Pear	E		0.111	0.040	0.1	2.6	0.325	0.025	0.325
Cam d	Camel milk	E		0.111	0.069	0.1	2.6	0.505	0.145	0.505
Bos d	Beef	E		0.127	0.130	0.1	2.6	0.88	0.21	0.88
nBos d 6	Beef	M	Serum albumin	0.208	0.812	0.1	2.6	4.03	3.17	4.03
Equ c	Horse meat	E		0.111	0.048	0.1	2.6	0.385	0.005	0.385
rSus d 1	Pork	M	Serum albumin	0.159	0.473	0.1	2.6	2.33	1.91	2.33
rAni s 3	Nematodes	M	Tropomyosin	0.523	3.576	0.1	2.6	16.185	15.295	16.185
Chi spp.	Crab	E		0.451	2.942	0.1	2.6	13.425	12.495	13.425
Lol spp.	Squid	E		0.188	0.637	0.1	2.6	3.065	2.625	3.065
Ost e	Oyster	E		0.166	0.512	0.1	2.6	2.47	2.12	2.47
nPen m 1	Tiger prawn	M	Tropomyosin	0.554	3.813	0.1	2.6	17.345	16.215	17.345
Rud spp.	Mollusc	E		0.230	1.031	0.1	2.6	4.735	4.415	4.735
rAlt a 6	Alternaria alternata	M	Enolase	0.107	0.043	0.1	2.6	0.365	0.055	0.365
rCan f 2	Dog	M	Lipocalin	0.163	0.499	0.1	2.6	2.47	2.0	2.47
rCan f Fel d 1 like	Dog	M	Secretoglobin	0.123	0.105	0.1	2.6	0.67	0.29	0.67
rCav p 1	Guinea pig	M	Lipocalin	0.108	0.052	0.1	2.6	0.41	0.1	0.41
rBos d 2	Cow epithelium	M	Lipocalin	0.154	0.323	0.1	2.6	2.105	0.235	2.105
rEqu c 1	Equine epithelium	M	Lipocalin	0.515	2.928	0.1	2.6	15.645	9.525	15.645
nEqu c 3	Equine epithelium	M	Serum albumin	0.187	0.721	0.1	2.6	3.375	3.055	3.375
Sus d	Pig epithelium	E		0.123	0.142	0.1	2.6	0.96	0.0	0.96
Cap h	Goat epithelium	E		0.158	0.343	0.1	2.6	2.155	0.605	2.155
Api m	Bee's venom	E		0.109	0.050	0.1	2.6	0.4	0.08	0.4
rBla g 4	Cockroach	M	Lipocalin	0.136	0.068	0.1	2.6	0.42	0.04	0.42
rPer a 7	American cockroach	M	Tropomyosin	0.492	3.297	0.1	2.6	15.005	14.025	15.005
rPhl p 12	Meadow timothy	M	Profilin	0.165	0.392	0.1	1.3	2.54	0.23	2.54
Phr c	Common reed	E		0.123	0.101	0.1	1.3	0.94	0.0	0.94
Amb a	Common ragweed	E		0.121	0.132	0.1	1.3	0.46	0.0	0.46
rAmb a 4	Common ragweed	M	Plant defensin	0.138	0.312	0.1	1.3	2.84	0.0	2.84
Pla l	Plantago lanceolata	E		0.107	0.062	0.1	1.3	0.64	0.0	0.64
rCan s 3	Hemp	M	nsLTP	0.106	0.046	0.1	1.3	0.5	0.0	0.5
rTri a 14	Common wheat	M	nsLTP	0.166	0.569	0.1	1.3	5.09	0.0	5.09
rTri a 19	Common wheat	M	Omega-5-Gliadin	0.113	0.037	0.1	1.3	0.3	0.0	0.3
Ory s	Rice	E		0.107	0.031	0.1	1.3	0.34	0.0	0.34
Zea m	Corn	E		0.108	0.058	0.1	1.3	0.59	0.0	0.59

rZea m 14	Corn	M	nsLTP	0.110	0.036	0.1	1.3	0.33	0.0	0.33
Pim a	Anise	E		0.123	0.135	0.1	1.3	1.27	0.0	1.27
rBet v 6	Silver birch	M	Isoflavone reductase	0.110	0.043	0.1	1.3	0.4	0.0	0.4
nOle e 1	Olive	M	Family Ole e 1	0.110	0.083	0.1	1.3	0.83	0.0	0.83
rOle e 9	Olive	M	1,3 β Glucanase	0.154	0.461	0.1	1.3	4.15	0.0	4.15
rPla a 3	Maple-leaved plane tree	M	NsLTP	0.114	0.120	0.1	1.3	1.15	0.0	1.15
Mor r	Red mulberry	E		0.108	0.032	0.1	1.3	0.32	0.0	0.32
Bro p	Paper mulberry	E		0.115	0.081	0.1	1.3	0.79	0.0	0.79
rBlo t 5	Blomia tropicalis	M	Mites group 5	0.163	0.547	0.1	1.3	4.9	0.0	4.9
rDer f 2	Dermatophagoides farinae	M	Family NPC2	0.125	0.219	0.1	1.3	2.02	0.0	2.02
rDer p 7	Dermatophagoides pteronyssinus	M	Mites group 7	0.106	0.046	0.1	1.3	0.5	0.0	0.5
rDer p 21	Dermatophagoides pteronyssinus	M	Unknown	0.113	0.094	0.1	1.3	0.92	0.0	0.92
Aca s	Acarus siro	E		0.127	0.218	0.1	1.3	2.01	0.0	2.01
rTry p 2	Tyrophagus putrescentiae	M	Family NPC2	0.111	0.038	0.1	1.3	0.37	0.0	0.37
rGly d 2	Glycyphagus domesticus	M	Family NPC2	0.164	0.553	0.1	1.3	4.95	0.0	4.95
rLep d 2	Lepidoglyphus destructor	M	Family NPC2	0.225	1.079	0.1	1.3	9.57	0.0	9.57
Pers a	Avocado	E		0.106	0.026	0.1	1.3	0.3	0.0	0.3
nAra h 9	Peanut	M	nsLTP	0.108	0.049	0.1	1.3	0.52	0.0	0.52
rCor a 8	Hazelnut	M	nsLTP	0.119	0.160	0.1	1.3	1.5	0.0	1.5
nBer e 1	Brazil nut	M	Albumin 2S	0.107	0.062	0.1	1.3	0.64	0.0	0.64
rPis v 1	Pistachio	M	Albumin 2S	0.121	0.179	0.1	1.3	1.67	0.0	1.67
nPis v 3	Pistachio	M	Globulin 7/8S	0.106	0.039	0.1	1.3	0.44	0.0	0.44
nPap s	Poppy seeds	M	Albumin 2S	0.107	0.062	0.1	1.3	0.64	0.0	0.64
rGly m 4	Soy	M	PR-10	0.105	0.039	0.1	1.3	0.43	0.0	0.43
nAct d 10	Kiwi	M	nsLTP	0.104	0.032	0.1	1.3	0.38	0.0	0.38
Fic c	Fig	E		0.121	0.074	0.1	1.3	0.69	0.0	0.69
nMal d 2	Apple	M	TLP	0.105	0.040	0.1	1.3	0.45	0.0	0.45
rMal d 3	Apple	M	nsLTP	0.119	0.164	0.1	1.3	1.54	0.0	1.54
rPru p 3	Peach	M	nsLTP	0.128	0.242	0.1	1.3	2.22	0.0	2.22
Vac m	Bilberry	E		0.119	0.073	0.1	1.3	0.69	0.0	0.69
nVit v 1	Grapes	M	nsLTP	0.110	0.073	0.1	1.3	0.72	0.0	0.72
Equ c	Mare's milk	E		0.107	0.062	0.1	1.3	0.64	0.0	0.64
Gal d	Egg yolk	E		0.125	0.157	0.1	1.3	1.47	0.0	1.47
nGal d 5	Egg yolk	M	Serum albumin	0.104	0.036	0.1	1.3	0.42	0.0	0.42
Ovi a	Sheep meat	E		0.123	0.196	0.1	1.3	1.82	0.0	1.82
rCyp c 1	Carp	M	β-parwalbumin	0.585	4.197	0.1	1.3	36.93	0.0	36.93
Gad m	Atlantic cod	E		0.106	0.047	0.1	1.3	0.51	0.0	0.51
nGad m 1	Atlantic cod	M	β-parwalbumin	0.112	0.104	0.1	1.3	1.01	0.0	1.01
rSal s 1	Salmon	M	β-parwalbumin	0.136	0.313	0.1	1.3	2.85	0.0	2.85
rThu a 1	Tuna	M	β-parwalbumin	0.588	4.222	0.1	1.3	37.15	0.0	37.15
Hom g	Lobster	E		0.302	1.736	0.1	1.3	15.34	0.0	15.34
Lit s	White shrimp	E		0.379	2.397	0.1	1.3	21.14	0.0	21.14
Pan b	Northern prawn	E		0.198	0.836	0.1	1.3	7.44	0.0	7.44
rPen m 4	Tiger prawn	M	Calcium-binding sarcoplasmic protein	0.180	0.695	0.1	1.3	6.2	0.0	6.2
rClu h 1	Atlantic herring	M	β-parwalbumin	0.113	0.090	0.1	1.3	0.87	0.0	0.87
Sco s	Atlantic mackerel	E		0.116	0.114	0.1	1.3	1.09	0.0	1.09
rSco s 1	Atlantic mackerel	M	β-parwalbumin	0.570	4.073	0.1	1.3	35.84	0.0	35.84
rXip g 1	Swordfish	M	β-parwalbumin	0.566	4.033	0.1	1.3	35.49	0.0	35.49
rAsp f 4	Aspergillus fumigatus	M	Unknown	0.159	0.435	0.1	1.3	3.92	0.0	3.92
Pen ch	Penicillium chrysogenum	E		0.111	0.043	0.1	1.3	0.36	0.0	0.36
nHev b 8	Latex	M	Profilin	0.149	0.417	0.1	1.3	3.76	0.0	3.76
rHev b 11	Latex	M	Class I chitinase	0.105	0.037	0.1	1.3	0.42	0.0	0.42

rArg r 1	European pigeon tick	M	Lipocalin	0.146	0.385	0.1	1.3	3.48	0.0	3.48
nCan f 3	Dog	M	Serum albumin	0.105	0.029	0.1	1.3	0.3	0.0	0.3
nFel d 2	Cat	M	Serum albumin	0.126	0.207	0.1	1.3	1.91	0.0	1.91
rFel d 4	Cat	M	Lipocalin	0.368	2.319	0.1	1.3	20.45	0.0	20.45
rPho s 1	Djungarian hamster	M	Lipocalin	0.260	1.355	0.1	1.3	11.99	0.0	11.99
Rat n	Rat	E		0.333	2.003	0.1	1.3	17.68	0.0	17.68
rVes v 1	Common wasp venom	M	Phospholipase A1	0.101	0.008	0.1	1.3	0.0	0.0	0.0
rVes v 5	Common wasp venom	M	Antigen 5	0.145	0.233	0.1	1.3	2.13	0.0	2.13
Per a	American cockroach	E		0.138	0.287	0.1	1.3	2.62	0.0	2.62
rPhl p 7	Meadow timothy	M	Polcalcin	<0.1	-	-	0	<0.1	-	-
rArt v 3	Mugwort	M	nsLTP	<0.1	-	-	0	<0.1	-	-
rAmb a 1	Common ragweed	M	Pectin lyase	<0.1	-	-	0	<0.1	-	-
Che a	White quinoa	E		<0.1	-	-	0	<0.1	-	-
rChe a 1	White quinoa	M	Family Ole e 1	<0.1	-	-	0	<0.1	-	-
Par j	Bricklayer	E		<0.1	-	-	0	<0.1	-	-
rPar j 2	Bricklayer	M	nsLTP	<0.1	-	-	0	<0.1	-	-
rPla l 1	Plantago lanceolata	M	Family Ole e 1	<0.1	-	-	0	<0.1	-	-
rSal k 1	Prickly brine	M	Pectin methylesterase	<0.1	-	-	0	<0.1	-	-
Can s	Hemp	E		<0.1	-	-	0	<0.1	-	-
rTri a aA_TI	Common wheat	M	α-amylase and trypsin inhibitor	<0.1	-	-	0	<0.1	-	-
Tri s	Spelled wheat	E		<0.1	-	-	0	<0.1	-	-
Hor v	Barley	E		<0.1	-	-	0	<0.1	-	-
nFag e 2	Buckwheat	M	Albumin 2s	<0.1	-	-	0	<0.1	-	-
Car c	Caraway seed	E		<0.1	-	-	0	<0.1	-	-
Ori v	Oregano	E		<0.1	-	-	0	<0.1	-	-
Pet c	Parsley	E		<0.1	-	-	0	<0.1	-	-
rAln g 4	Black alder	M	Polcalcin	<0.1	-	-	0	<0.1	-	-
rPla a 1	Maple-leaved plane tree	M	Plant invertase	<0.1	-	-	0	<0.1	-	-
Fra e	Common ash	E		<0.1	-	-	0	<0.1	-	-
rFra e 1	Common ash	M	Family Ole e 1	<0.1	-	-	0	<0.1	-	-
rBlo t 21	Blomia tropicalis	M	Unknown	<0.1	-	-	0	<0.1	-	-
rDer p 11	Dermatophagoides pteronyssinus	M	Myosin heavy chain	<0.1	-	-	0	<0.1	-	-
rDer p 20	Dermatophagoides pteronyssinus	M	Arginine kinase	<0.1	-	-	0	<0.1	-	-
Tyr p	Tyrophagus putrescentiae	E		<0.1	-	-	0	<0.1	-	-
rApi g 2	Celery	M	nsLTP	<0.1	-	-	0	<0.1	-	-
rApi g6	Celery	M	nsLTP	<0.1	-	-	0	<0.1	-	-
nSola l 6	Tomato	M	nsLTP	<0.1	-	-	0	<0.1	-	-
nJug r 3	Walnut	M	nsLTP	<0.1	-	-	0	<0.1	-	-
rAna o 2	Cashew nut	M	Globulin 11S	<0.1	-	-	0	<0.1	-	-
nPis v 2	Pistachio	M	Globulin 11S subunit	<0.1	-	-	0	<0.1	-	-
rGly m 5	Soy	M	Globulin 7/8S	<0.1	-	-	0	<0.1	-	-
rGly m 8	Soy	M	Albumin 2S	<0.1	-	-	0	<0.1	-	-
nAct d 5	Kiwi	M	Kiwellin	<0.1	-	-	0	<0.1	-	-
Cit s	Orange	E		<0.1	-	-	0	<0.1	-	-
Man i	Mango	E		<0.1	-	-	0	<0.1	-	-
Pru av	Cherry	E		<0.1	-	-	0	<0.1	-	-
Gal d	Chicken meat	E		<0.1	-	-	0	<0.1	-	-
Mel g	Turkey meat	E		<0.1	-	-	0	<0.1	-	-
Ory c	Rabbit meat	E		<0.1	-	-	0	<0.1	-	-
Sus d	Pork	E		<0.1	-	-	0	<0.1	-	-
nGad m 2+3	Atlantic cod	M	β-enolase + Aldolase	<0.1	-	-	0	<0.1	-	-

Sal s	Salmon	E		<0.1	-	-	0	<0.1	-	-
Thu a	Tuna	E		<0.1	-	-	0	<0.1	-	-
rAni s 1	Nematodes	M	Kunitz-type serine protease inhibitor	<0.1	-	-	0	<0.1	-	-
Myt e	Common mussel	E		<0.1	-	-	0	<0.1	-	-
rPen m 2	Tiger prawn	M	Arginine kinase	<0.1	-	-	0	<0.1	-	-
rPen m 3	Tiger prawn	M	Myosin light chain	<0.1	-	-	0	<0.1	-	-
Pec spp.	Scallop	E		<0.1	-	-	0	<0.1	-	-
Clu h	Atlantic herring	E		<0.1	-	-	0	<0.1	-	-
rCra c 6	Common shrimp	M	Troponin C	<0.1	-	-	0	<0.1	-	-
Raj c	Spiny stingray	E		<0.1	-	-	0	<0.1	-	-
rRaj c Parw.	Spiny stingray	M	α-parvalbumin	<0.1	-	-	0	<0.1	-	-
rAsp f 1	Aspergillus fumigatus	M	Mitogyllin family	<0.1	-	-	0,0	<0.1	-	-
rAsp f 6	Aspergillus fumigatus	M	Mitochondrial superoxide dismutase	<0.1	-	-	0	<0.1	-	-
Cla h	Cladosporium herbarum	E		<0.1	-	-	0	<0.1	-	-
rCla h 8	Cladosporium herbarum	M	Short-chain dehydrogenase	<0.1	-	-	0	<0.1	-	-
rMala s 5	Malassezia sympodialis	M	Unknown	<0.1	-	-	0	<0.1	-	-
rHev b 1	Latex	M	REF (rubber elongation factor)	<0.1	-	-	0	<0.1	-	-
rHev b 3	Latex	M	SRPP (small rubber particle protein)	<0.1	-	-	0	<0.1	-	-
rHev b 5	Latex	M	Unknown	<0.1	-	-	0	<0.1	-	-
rHev b 6.02	Latex	M	Proheweina	<0.1	-	-	0	<0.1	-	-
Fic b	Ficus	E		<0.1	-	-	0	<0.1	-	-
Sac c	Baker's yeast	E		<0.1	-	-	0	<0.1	-	-
rOry c 1	Rabbit epithelium	M	Lipocalin	<0.1	-	-	0	<0.1	-	-
rOry c 2	Rabbit epithelium	M	Lipophilin	<0.1	-	-	0	<0.1	-	-
rEqu c 4	Equine epithelium	M	Laferin	<0.1	-	-	0	<0.1	-	-
Ovi a	Sheep epithelium	E		<0.1	-	-	0	<0.1	-	-
nApi m 1	Bee's venom	M	Phospholipase A2	<0.1	-	-	0	<0.1	-	-
rApi m 10	Bee's venom	M	Icarpaina var. 2	<0.1	-	-	0	<0.1	-	-
Pol d	Redhorn beetle venom	E		<0.1	-	-	0	<0.1	-	-
rPol d 5	Redhorn beetle venom	M	Antigen 5	<0.1	-	-	0	<0.1	-	-
Ves v	Common wasp venom	E		<0.1	-	-	0	<0.1	-	-
Dol spp.	Hornet venom	E		<0.1	-	-	0	<0.1	-	-
rBla g 1	Cockroach	M	Cockroaches group 1	<0.1	-	-	0	<0.1	-	-
rBla g 2	Cockroach	M	Aspartyl protease	<0.1	-	-	0	<0.1	-	-
rBla g 5	Cockroach	M	Glutathione S-transferase	<0.1	-	-	0	<0.1	-	-
rBla g 9	Cockroach	M	Arginine kinase	<0.1	-	-	0	<0.1	-	-

Table S2. Results of Shapiro-Wilk test.

Amino Acid	Abbreviation	*p* value Shapiro-Wilk Test		
		IgE-Mediated Allergy Group	Non-IgE-Mediated Allergy Group	Control Group
1-Methyl-L-histidine	1MHis	<0.001	0.065	<0.001
3-Methyl-L-histidine	3MHis	<0.001	0.002	<0.001
L-α-Aminoadipic acid	Aad	0.004	0.918	<0.001
L-α-Amino-n-butyric acid	Abu	0.063	0.015	0.852
L-Alanine	Ala	0.502	0.560	0.605
L-Arginine	Arg	0.418	0.011	0.490
L-Asparagine	Asn	<0.001	0.958	0.801
L-Aspartic acid	Asp	0.161	0.958	0.241
D,L-β-Aminoisobutyric acid	bAib	<0.001	0.033	0.160
β-Alanine	bAla	<0.001	0.218	<0.001
L-Citrulline	Cit	0.546	0.003	0.412
Ethanolamine	EtN	0.032	0.083	0.142
γ-Amino-n-butyric acid	GABA	0.006	0.001	0.004
L-Glutamine	Gln	0.463	0.189	0.339
L-Glutamic acid	Glu	0.115	0.152	0.485
Glycine	Gly	0.484	0.327	0.007
Homocitrulline	Hcit	0.018	0.003	0.024
L-Histidine	His	<0.001	0.656	0.528
Hydroxylysine	Hyl	<0.001	0.461	<0.001
Hydroxy-L-proline	Hyp	<0.001	0.083	0.001
L-Isoleucine	Ile	<0.001	0.360	0.343
L-Leucine	Leu	0.005	0.551	0.240
L-Lysine	Lys	0.002	0.189	0.253
L-Methionine	Met	<0.001	0.217	0.174
L-Ornithine	Orn	0.008	0.122	0.940
L-Phenylanalanine	Phe	0.022	0.017	0.872
L-Proline	Pro	0.117	0.333	0.355
Sarcosine	Sar	0.002	<0.001	0.424
L-Serine	Ser	0.324	0.860	0.162
Taurine	Tau	0.16	0.996	0.028
L-Threonine	Thr	0.006	0.228	0.604
L-Tryptophan	Trp	0.017	0.983	0.009
L-Tyrosine	Tyr	<0.001	0.036	0.311
L-Valine	Val	0.001	0.867	0.447
